# On the ethical and moral dimensions of using artificial intelligence for evidence synthesis

**DOI:** 10.1371/journal.pgph.0004348

**Published:** 2025-03-19

**Authors:** Soumyadeep Bhaumik

**Affiliations:** 1 Meta-research and Evidence Synthesis Unit, Health Systems Science, The George Institute for Global Health, Sydney, Australia; 2 Faculty of Medicine and Health, UNSW Sydney, Kensington, Australia; 3 The George Institute for Global Health, New Delhi, India; 4 Department of Public Health, Walter Sisulu University, Mthatha, South Africa; Islamic Azad University South Tehran Branch, IRAN, ISLAMIC REPUBLIC OF

## Abstract

Artificial intelligence (AI) is increasingly being used in the field of medicine and healthcare. However, there are no articles specifically examining ethical and moral dimensions of AI use for evidence synthesis. This article attempts to fills this gap. In doing so, I deploy in written form, what in Bengali philosophy and culture, is the *Adda* (আড্ডা) approach, a form of oral exchange, which involves deep but conversational style discussion. Adda developed as a form of intellectual resistance against the cultural hegemony of British Imperialism and entails asking provocative question to encourage critical discourse.The raison d’être for using AI is that it would enhance efficiency in the conduct of evidence synthesis, thus leading to greater evidence uptake. I question whether assuming so without any empirical evidence is ethical. I then examine the challenges posed by the lack of moral agency of AI; the issue of bias and discrimination being amplified through AI driven evidence synthesis; ethical and moral dimensions of epistemic (knowledge-related) uncertainty on AI; impact of knowledge systems (training of future scientists, and epistemic conformity), and the need for looking at ethical and moral dimensions beyond technical evaluation of AI models. I then discuss ethical and moral responsibilities of government, multi-laterals, research institutions and funders in regulating and having an oversight role in development, validation, and conduct of evidence synthesis. I argue that industry self-regulation for responsible use of AI is unlikely to address ethical and moral concerns, and that there is a need to develop legal frameworks, ethics codes, and of bringing such work within the ambit of institutional ethics committees to enable appreciation of the complexities around use of AI for evidence synthesis, mitigate against moral hazards, and ensure that evidence synthesis leads to improvement of health of individuals, nations and societies.

## Introduction

Artificial intelligence (AI) is a computational system designed to imitate tasks which intelligent beings can conduct. AI systems are capable of drawing inferences and engage in “unsupervised dynamic learning” from data. AI has been deployed globally with little regulatory or ethical oversight - leading to governance of AI playing catch up with its deployment.[1] Despite the rapid pace of change, scholars have examined the ethics of using AI in medical and health research from various angles. [2–10] While AI is being increasing used for evidence synthesis in health and medicine, [11, 12] there are no articles examining ethical and moral dimensions of AI use for conducting evidence synthesis. This article attempts to fill this gap.

In doing so, I deploy in written form, what in Bengali philosophy and culture, is the *Adda* (আড্ডা) approach.^1^ *Adda* is a form of oral intellectual exchange, which involves conversational style, yet deep and critical discussions. It involves laying down one’s viewpoints and asking provocative questions to encourage diverse critical discourse. *Adda* originated in Bengal (now divided between Bangladesh and India) as a form of resistance by the urban working Bengali against the cultural hegemony of British imperialism – it has an element of performativity.[13] An *Adda* session is rarely conclusive in nature. It raises more questions than what initially existed, thereby fostering ongoing inquiry and reflection.

### The raison d’être for using AI for evidence synthesis

Evidence synthesis, irrespective of its type, typically involves three steps: retrieving evidence, comparing evidence, and synthesising evidence. [14] The synthesis is done in a manner which allows evidence users (policy makers, practitioners, researchers patients and public) to make informed decisions. Due to its inherent value in the knowledge translation pathway its efficient and accurate conduct is of immense importance.[15] But the conduct of high-quality evidence synthesis is a labour and time intensive process: requiring months to complete. [16] It is envisaged that AI systems by improving efficiency in the conduct of evidence synthesis, would lead to greater evidence uptake, and consequently improving health outcomes. This is the the raison d’être for use of AI in conducting evidence synthesis.

But is it ethical to not empirically evaluate the basic assumptions underlying this *raison d’être*? Is efficiency and speed the major factor impeding uptake of evidence? Would (and what kind of) evidence users trust an AI enabled evidence synthesis?

### Lack of moral agency

Although AI can imitate tasks performed by intelligent being, it does not have moral agency.[4,17] The lack of consciousness, values, life experiences, emotions, and meaningful connection to our physical universe raises two distinct, but related, issues:

- How do we prevent AI model to generate outputs, which are morally wrong or harmful?
- Who will be morally accountable for evidence synthesis outputs generated from AI models?

Apart from building in technical safeguards against harmful advice(which we discuss in subsequent section) in AI algorithms, the challenge of lack of moral agency of AI systems, is overcome through:

- by putting the responsibility and accountability to humans using AI for conducting evidence synthesis.
- human-centric modality of deployment (that is steps in evidence synthesis being AI enabled, rather than being fully automated).

The approach of fixing accountability to humans is legally tenable. Our entire civil and criminal justice system is built around the assumption that humans (with moral agency) are responsible for decision making.[18] But one might ask, if it is fair to hold a person legally accountable for mistakes done by a machine? Accountability after all is related to moral engagement while executing a task. [19] With AI systems eventually becoming capable of doing all tasks without involving humans, is it not prudent to hold those who develop AI systems and tools, or validate them accountable?

An AI-driven fully automated future of evidence synthesis is not entirely bad. The democratisation of health information, wherein patients conduct their own evidence synthesis will empower patients. It will mitigate the current power imbalance between patients, healthcare providers, and researchers.[20] But it is essential to ask, if our society has safeguards in place to prevent self-intervention based on AI driven evidence synthesis? Would the availability of such tools selectively benefit health of few groups, furthering health disparities?

### Bias and discrimination

AI systems are known to produce results which reflect and perpetuate human biases[4,21] due to several technical reasons:

- AI systems learn from data they have been trained on. These systems developed in the western world use training data which are typically representative of historical discriminations and framing of health issues of non-majoritarian groups in a stigmatising manner. The AI replicates these biases in its outputs.
- Bias might also arise due to the way developers have labelled data or excluded data – reflecting cognitive bias of developers.
- The design of the AI algorithm might be biased due to biased decisions taken by developers.
- There might be biased deployment of safeguards in different geographies, mirroring discriminatory bias of humans developing the AI system or tools developed from AI systems.

Examples of such bias from primary research, include but are not limited to, missed diagnoses of skin cancer in darker-skinned patients, and greater allocation of health resources to White patients over Black patients, despite similar health needs.[22,23]

Are safeguards in place to prevent amplification of such discriminatory biases in evidence synthesis? The opacity of training datasets, and AI algorithms makes assessment of such safeguards difficult for both those developing tools from AI systems, and evidence synthesis specialists.

It begets further questions. Is it ethical to use AI tools for evidence synthesis until such appropriate safeguards are available? Who is responsible for evaluating the appropriateness of safeguards?

### Epistemic uncertainty

Many researchers are working to understand the validity, safety, and accuracy of AI tools for conducting evidence synthesis. But at this point there are several epistemic (knowledge-related) uncertainties.[24] It puts evidence synthesis specialists in an ethical dilemma. Should we refrain from using AI for evidence synthesis outside of development and validation studies, or given the inefficiencies in the ways evidence synthesis is conducted currently, should we start deploying AI? The former stance aligns with the “do no harm” principle. The latter stance accepts that some epistemic uncertainty is inevitable for any new technology.

Some interest-holders might navigate this dilemma by using the exhaustive alternatives principle. The principle implies until AI tools have sufficient level of technical accuracy, validity, and safeguards, their use would be unethical unless all other viable alternatives of conducting evidence synthesis have been exhausted. In practical terms, the use of AI would be permissible only when evidence synthesis is linked to a specific time-sensitive decision-making process. Even in such circumstances, rapid evidence synthesis, a well-established approach for evidence synthesis to inform health policy and system decision making already exists. [25] For interest-holders willing to accept epistemic uncertainty, it might be prudent to ask, “What degree of uncertainty is acceptable for deployment beyond validation and testing studies?”

### Impacts on knowledge system

“If I have seen further, it is by standing on the shoulders of giants,” said Issac Newton, drawing an analogy of how novel scientific ideas are built on existing knowledge.

Critical analysis of existing knowledge has been part of scientific tradition in every culture. The development of evidence synthesis, as a research methodology in the last three decades has made it systematic, and less prone to bias. As such, doctoral programs to create the future generation of medical and health scientists, usually require conduct of some form of evidence synthesis. The use of AI tools for evidence synthesis, changes this paradigm. It changed the essence of how trainee scientists engage with existing knowledge. The critical engagement through reading, inferring, and extracting data which had to be synthesised using appropriate methods would be replaced by the technical act of navigating an AI software. Over time, the AI software will inherently transition to being a “black box” for trainee scientists. What would this mean for the future of science?

Such changes would also make human knowledge prone to epistemic conformity. Epistemic conformity refers to the tendency of AI systems to reinforce concept, ideas, and frameworks from dominant knowledge systems. [26] Further countries, universities, and research groups with access to greater computing power, would be able to dominate the global literature by volume – entrenching further the power accumulated to the societal positioning as knowledge generators and gatekeepers. This dominance might lead to research evidence being synthesised in a singular, monotheistic fashion, without critical engagement from individuals who interact with the physical world where research is conducted, and where the evidence will be applied. Should we consider AI a “weapon of mass destruction” of knowledge systems, with the potential of precipitating epistemic genocide?

One might argue that these risks would not exist if AI use were limited to the step of retrieving evidence. Even limiting use of AI in retrieving evidence by using the “researcher in the loop” modality of deployment offers huge efficiency gains. [12,27] It is a valid argument. It mitigates negative impacts on knowledge systems significantly, but not in entirety. Being aware of, and engaging with evidence not meeting inclusion criteria is an essential part of critical enquiry which evidence synthesis entails.

### Need to go beyond technical evaluation of AI models

People perceive AI as a tool, owing to its individualised interface. In reality, it is a large socio-technical system where several actors contribute by providing input data, design, testing, validation, and deployment.[28] It is therefore important to not only evaluate the AI model for technical validity and fairness, but also the relationships through which an AI model is developed, implemented, and owned. Industries which are traditionally deemed to harm public health interests (tobacco, alcohol, weapon-manufacturing, and fossil fuel industries) [29] might have investments and control over AI systems, or simply be clients. As for example, WHO Europe notes that, “ corporations are collaborating with technology giants to integrate health-harming products and brands seamlessly into our digital lives and culture.”[30] Would AI be an avenue to seamlessly integrate interests of health-harming industries in the knowledge translation pathway? The data provided for conducting evidence synthesis, might also train the AI system for digital targeting of breast-milk substitutes, tobacco, and alcohol. Worse still, it might also train AI systems for operating drones to target food and water supplies during war.

It is also essential to recognise that AI technologies have multiple uses. It is likely what the AI model learns through evidence synthesis of medicine and health data is used for other purposes. This aspect of AI is worrisome because of the potential for causing overall material risk of harm, including for warfare. As for example, Google in February 2025, removed[31] its earlier pledge to not use AI for “weapons or other technologies whose principal purpose or implementation is to cause or directly facilitate injury to people.”[32]

This leads us to a critical ethical question, “Is it ethically and morally acceptable to use AI systems for conducting health and medical research, when it has a risk for harming health?”

### Ethical and moral responsibility of governments and multi-lateral organisations

Considering the multitude of ethical and moral dimensions of AI use in evidence synthesis, governments are responsible for conducting industry-independent bias assessment, monitoring algorithms, developing frameworks for assessment, and delivering on appropriate legal frameworks. Additionally, bad faith actors (individuals or organised group) might be deliberately building in discriminatory bias while developing AI tools for evidence synthesis, or in future take advantage of vulnerability in AI systems. There is no reason to think AI systems will not be tweaked by bad faith actors to enable bias which can enable profits or build popular support of hate crimes against minorities.

It is worthwhile recalling that AI tools would in future be used by patients, and health workers to conduct their own evidence synthesis. This makes it imperative to see AI tools for evidence synthesis as medical devices – they provide health information, which influences clinical decisions. Path dependency for technology implies the need for building in regulations now, while the technology is still in its infancy with limited number of actors driving it, rather than later when the technology is too entrenched.

It is expected that the AI industry and norm-setting evidence synthesis organisations will engage in self-regulation by developing good practice standards. This is not sufficient because they bear inherent bias and conflicts (commercial and epistemic). They are also unlikely to be able to look at things granularly, such that standards are culturally sensitive, and contextually appropriate in the setting in which evidence synthesis is being conducted, how it will be used, and their impact on local knowledge ecosystems. As for example, the first such set of 8 principles guiding automation of systematic reviews by the International Collaboration for the Automation of Systematic Reviews (ICASR) completely overlooks ethical, and moral aspects focussing only on technical aspects of evidence synthesis.[33] The 2024 public draft of **R**esponsible **AI** in **E**vidence **S**ynth**E**sis (RAISE v.0.9) available for public consultation did not identify institutional ethics committees or government regulators as an interest-holder, despite the documents purportedly aim to develop standards to ensure integrity of research.[34] This is akin to industry self-regulation measures seen in other sectors which create the notion of morality and responsibility to avoid government and multi-lateral regulation to protect individual, communities, and nations. The need is for governments to regulate development of AI tools for conducting evidence synthesis. However, the cross-jurisdictional nature of both the AI and evidence synthesis ecosystem makes effective regulation challenging for national governments.

These raises a question: Is it not the ethical responsibility of governments and multilaterals(like WHO) to develop a legal framework for ethical governance of AI systems in medical and health research, which is inclusive of its use in evidence synthesis?

### Ethical responsibility of research institutions and funders

Given the myriads of ethical dimensions of using AI for evidence synthesis, research institutions and funders have a moral responsibility to have an oversight on:

- research to develop AI-tools for evidence synthesis (proof-of-concept and validation studies),
- evidence synthesis using prompts in generic AI tools(potential for cognitive and discriminatory bias due to framing of prompts)
- using AI system for conducting evidence synthesis or developing AI tools.

Such an oversight will safeguard research institutions and funders against moral hazards. There are also potential legal hazards in the future when patients conduct evidence synthesis using tools. Copyright and data sovereignty are also issues to considers. Additionally, medical and health research institutions and funders have code of conducts around engagement with harmful to health industries.

Considering the multi-use nature of AI models, and the potential of harms to health (and use in warfare), I ask, whether it is unethical if the AI industry is not brought under the same code?

## Conclusion

It is essential to remember that ultimate raison d’être for use of AI for evidence synthesis is improving health. This implies consideration of factors beyond technical validity and efficiency. Moral and ethical dimensions of AI are of key importance for everyone involved in health and medical research, including evidence synthesis. I have attempted to discuss the complexity of ethical and moral issues around using AI for evidence synthesis and pitched for an intergenerational ethics approach. Recommendations for consideration by policy makers, research institutions/funders and researchers are presented in Box 1. Greater recognition of these issues in the medical research and evidence synthesis community, to which I belong, is essential. It is important to recognise that using AI is an act of creating space for a quasi-social actor in our community, it changes its social fabric.[35]

Technology, much like a cat, cannot be put back in the bag once it is out. Let us put a bell to ensure that the cat does not run wild and remains loved by all.

**Box 1: Responsible AI for evidence synthesis: Recommendations for consideration by policy makers, research institutions, researchers and funders**

- Develop multi-lateral legal framework on use of AI for medical and health research, inclusive of evidence synthesis
- Governments should consider regulating AI tools for evidence synthesis by developing specific regulations or bring them under those pertaining to medical devices.
- Revise existing standards and guidelines on medical and health research to be inclusive of AI for evidence synthesis
- Research developing or validating AI tools for evidence synthesis as well as any evidence synthesis using AI should be brought under the ambit of institutional ethics committee
- Evaluate AI systems and models (when choosing for evidence synthesis) beyond their technical aspects to include ownership, clientele, and harmful use beyond primary purpose. Research institutions and funders should develop whitelists of allowed AI systems based on such evaluation.
- Raise awareness and support scholarly work (case studies and empirical research) on ethical and moral dimensions of using AI for evidence synthesis – these should take an intergenerational view to build safeguards for the future.

## Footnotes

1 I acknowledge that Adda is difficult to replicate in a written form. I have taken this decolonial approach and tried to stay true to the spirit of Adda by structuring each section with context of the argument, conversational style laying down of viewpoint, and finally posing questions for others (and myself) to ponder. I welcome readers to critique in the written manner or join me for a true session of *Adda.*
